# The Influence of the Gradient Infill of PLA Samples Produced with the FDM Technique on Their Mechanical Properties

**DOI:** 10.3390/ma15041304

**Published:** 2022-02-10

**Authors:** Joanna Maszybrocka, Michał Dworak, Grażyna Nowakowska, Patrycja Osak, Bożena Łosiewicz

**Affiliations:** Institute of Materials Engineering, Faculty of Science and Technology, University of Silesia in Katowice, 75 Pułku Piechoty 1A, 41-500 Chorzow, Poland; michal.dworak@us.edu.pl (M.D.); g.nowakowska94@gmail.com (G.N.); patrycja.osak@us.edu.pl (P.O.)

**Keywords:** 3D printing, FDM technique, PLA, porosity, compressing

## Abstract

Three-dimensional printing is a dynamically developing field of industry. Its main advantage is the small amount of waste, no need to use specialized tools, and easy control of the mechanical properties of the printed model. One of the most popular techniques of 3D printing is FDM. The main factor influencing the mechanical properties of 3D-printed materials is the filling density. The aim of this study was to determine the mechanical properties of porous structures with a porosity gradient of PLA samples printed using the FDM technique. The accuracy of mapping the structures by computed tomography was assessed, and then a static compression test was performed. It has been shown that the strength properties increased with the increase in the filling density. The highest value of compression strength, amounting to 41.2 MPa, was observed for samples made of PLA with an 80% filling degree, whereas the lowest value of compression strength was found in PLA-T samples with a filling degree of 10%, reaching only 0.6 MPa. It was found that not only the core filling density, but also the outer layers, influences the mechanical properties. The assessment of spatial architecture allowed for a qualitative and quantitative assessment. The obtained images from the computed tomograph showed that the designed sample models were correctly reproduced in the entire volume.

## 1. Introduction

Three-dimensional printing is considered to be the foundation of the fourth industrial revolution. Its dynamic pace of development in recent years has changed the current view on modern production methods [1]. Contrary to traditional techniques, where the significant disadvantage is a large amount of waste, additive methods (AMs) generate much less waste. Numerous AMs make it possible to produce an element practically in one cycle (“at a time”), which is difficult to obtain with traditional techniques. The obtained details are characterized by different mechanical properties depending on the material type or the process parameters used. The basis of modern AMs is the formation of a product on the basis of a virtual model developed in computer systems [2,3,4,5]. The most popular AMs are stereolithography (SLA) [3], laminated object manufacturing (LOM) [5], selective laser melting (SLM) [6,7,8,9], PolyJet [10], three-dimensional printing (3DP) [10], and fused deposition modeling (FDM) [11,12,13,14,15,16,17,18].

The FDM technique, despite numerous limitations, is easy to use and the cheapest of all AMs. This method uses thermoplastics, such as acrylonitrile butadiene styrene (ABS) or polylactide (PLA), in the form of a line wound on a spool [13]. The material is fed to the head where it is heated to a specific temperature. The plasticized material is extruded through a die, which moves along the *x* and *y* axes, onto the movable table, which usually moves along the *z* axis [14]. After the appropriate layer of material is applied to the table, it is lowered by the thickness of the layer, and the next layer is deposited until the element is completely produced. In the preparation of complex elements, supports that can be removed are required.

Depending on the parameters of the FDM process, different properties of the finished part can be obtained [15]. For example, depending on the orientation of the model on the work platform, the finished part shows the anisotropy of mechanical properties [16]. The orientation of the model affects not only the mechanical properties and surface quality, but also the economic aspect. Parts placed vertically and at an angle on the platform print slower and require the use of supports, which increases the material consumption. The main factor influencing the mechanical properties of the printed part is the filling density. Depending on the potential application of the produced detail, the filling density can be reduced or increased. Reducing the filling density allows for less material consumption, but the manufactured details will be characterized by worse strength properties [17]. The printing speed is also an important parameter. Research shows that with the increasing printing speed, the strength properties, i.e., impact strength, hardness and tensile strength, decrease. The continuity of filling affects the accuracy and quality of the surface of the finished product. When it is disturbed, cavities may form, which will lower the mechanical properties of the model. By controlling the degree of filling, the stiffness of the finished model can be tailored. Changing the density of the filling also helps to reduce some of the production costs through lower material consumption and a shorter production time [3].

The impact of the degree and method of filling the structure on the properties of the FDM-printed model and, therefore, on the obtained strength parameters of the manufactured object, was the subject of research in [11]. The authors tested PLA samples with 20%, 35%, 50%, 65% and 80% filling. It was found that the compressive strength increased linearly with an increase in the density of the filling [11]. The maximum compressive strength of 30 MPa was obtained for the samples with the highest filling density, which was 80%. As the filling density increases, the time necessary to print such a model also increases. This is the result of the longer path the nozzle has to travel to print the same element.

In recent years, there has been a noticeable increase in interest in gradient filling of samples with high application potential. The combination of 3D printing with a gradient method of filling with an FEM strength analysis, can help to design structures that are sufficiently durable, with an economical way of using the material at the same time [19,20]. In [19], the authors proposed an algorithm that allows for a precise, local change of the degree of filling based on the distribution of stress values obtained on the basis of FEM simulation. The authors confirmed the effectiveness of the proposed solution experimentally. The obtained results indicate a 30% increase in flexural strength in the case of the sample with optimized packing, compared to the reference sample. Moreover, in the case of optimized samples, a greater reproducibility of the obtained results was noticed.

Three-dimensional printing shapes the development of many industries because it allows for the production of models of any shape, which is often not feasible using subtractive methods [21]. The combination of AMs with a wide range of building materials allows for specific mechanical properties. In AMs, the cell structures are characterized by a topology with a repeat unit cell. Such structures are characterized by a lower density while maintaining high strength properties. In their work [21], the authors assessed the influence of the material and structure topology on the deformation process and the ability to absorb plastic deformation energy under quasi-static loading conditions. The samples with honeycomb topology and its derivatives were tested. The samples were printed using the FDM method from three materials, i.e., ABS, polycarbonate (PC-10) and polyamide (Nylon 12). The ratio of plastic strain energy to the relative density of the structure was adopted as the criterion for assessing the impact of the structure topology on the ability to absorb energy. The test results show that the stiffness of the structure is determined by the strength of the material. The ability to absorb energy depends primarily on the topology used.

The wide possibilities offered by the production of cell structures by AMs are used more and more often in medicine for the production of cell scaffolds. With the help of 3D printing, the production process of such structures can be controlled, ensuring the appropriate pore architecture and shape depending on their intended use. The authors of [22] assessed the possibility of producing scaffolds using the FDM method from PLA. Nine variants of samples were analyzed, which differed in the degree of filling (60, 80, 100%), layer thickness (0.150, 0.250, 0.350 mm) and the method of filling (triangular, rectangular, honeycomb). It was found that the parameters of the printing process significantly affect the compressive strength of the tested samples. The authors postulate that this effect is related to the porosity of the samples.

Based on the review of the bibliography, it can be noted that most authors focus on the assessment of the impact of printing parameters, i.e., raster angle, build orientation, infill pattern, layer thickness, print temperature, bed temperature, etc., on the mechanical properties of the samples produced by the FDM method. The issues related to the production and mechanical properties of samples with a gradient filling (horizontal and vertical) produced by the FDM method are discussed much less frequently. Meanwhile, the use of variable filling density provides a wide range of control for the mechanical properties, thanks to which it is possible to achieve satisfactory strength with the lowest material consumption and ensuring that the structure is light.

The main objective of this study was to evaluate the mechanical properties of porous structures with a porosity gradient produced using FDM technology in the uniaxial compression test. The scope of the work included the development of a simple methodology for modeling controllable structures with a radial porosity gradient and preparation of models for printing, preparation on an Ultimaker 2+ printer, printing a series of PLA samples, an assessment of the internal architecture of the structure using the computed tomography method, and assessment of their mechanical properties in a static uniaxial compression test.

## 2. Materials and Methods

### 2.1. Material Chemical Composition

The samples were made of PLA and PLA Tough (T) (Spectrum Group, Pęcice, Poland). PLA is an aliphatic thermoplastic polymer that is obtained from raw materials of natural origin, such as corn meal. It is biodegradable and available in many color variants. PLA has good physical and rheological properties, similar to polyethylene terephthalate and polystyrene. Due to its non-toxicity, biodegradability and biocompatibility, PLA can be used in many industries as well as in medicine in biomedical applications [23,24,25].

**Figure 1:**
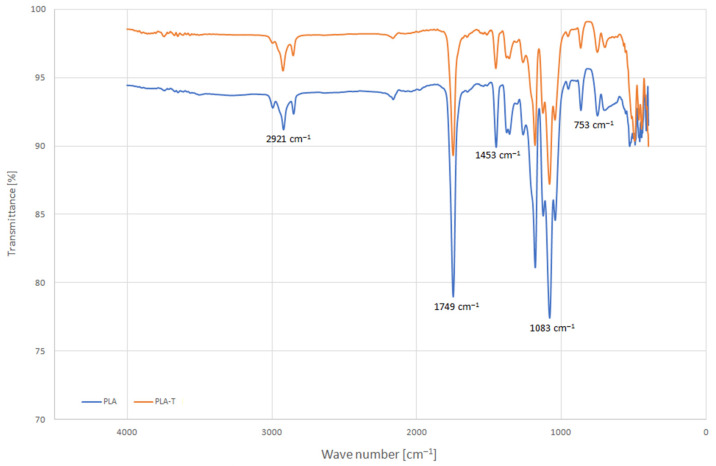
The ATR-FTIR absorption spectrum of PLA and PLA-T.

The chemical bonds in the PLA and PLA-T were determined by the attenuated total reflection—Fourier transform infrared spectroscopy (ATR-FTIR) using the Shimadzu IR Prestige-21 FTIR spectrophotometer equipped with an ATR attachment, which is described in more detail in the work [26]. Figure 1 shows the ATR-FTIR absorption spectra of PLA and PLA-T filaments. The band at 1749 cm^−1^ corresponds to the stretching vibration of the carbonyl groups of the polylactide. The ATR-FTiR spectrum shows the peaks characteristic for PLA at 2995–2851 cm^−1^, 1453 cm^−1^ and 1380 cm^−1^, corresponding to symmetric and asymmetric stretching vibrations of the CH_3_ group [27]. The band at 1183 cm^−1^ and three bands at 1128 cm^−1^, 1083 cm^−1^ and 1045 cm^−1^ are assigned to the stretching vibrations in the groups –C–O–, –CH–O– and –CO–O–, respectively, in PLA chains. The bands at 867 cm^−1^ and 753 cm^−1^ can be assigned to the amorphous and crystalline phases of PLA, respectively [28]. The ATR-FTIR spectrum obtained with the PLA-T shows the same absorption peaks as PLA without addition. This means that despite the reinforced filament, no new bond is formed, and no strong chemical interaction takes place [29].

### 2.2. Methodology of Modeling PLA Samples

**Figure 2:**
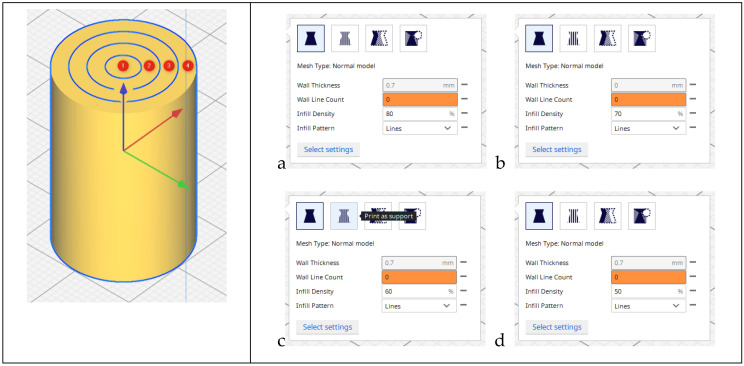
Setting the filling of individual zones: (**a**) 80% infill density; (**b**) 70% infill density; (**c**) 60% infill density; (**d**) 50% infill density.

**Figure 3:**
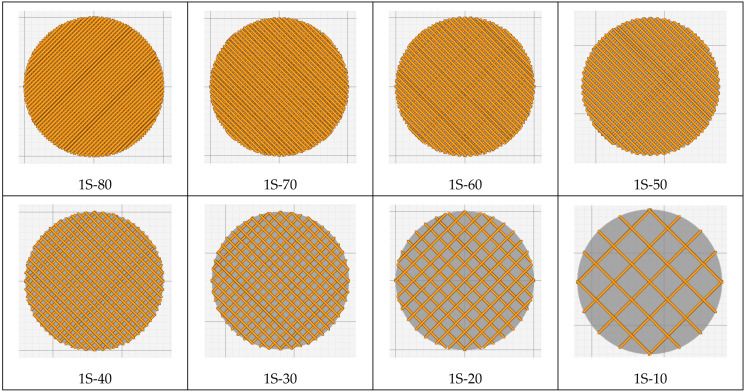
Visualization of the generated models with one zone (top view).

**Figure 4:**
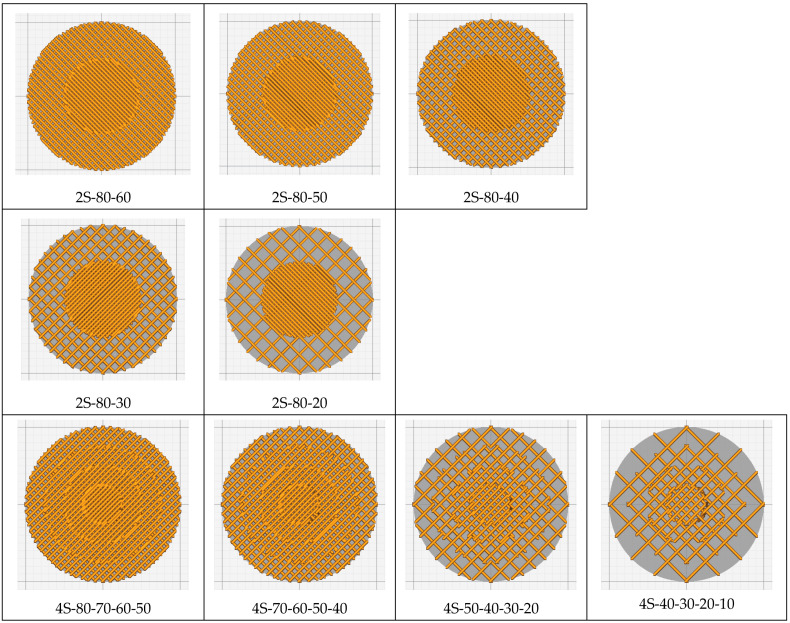
Visualization of the generated models with two and four zones (top view).

The sample model was prepared in the Fusion 360° program. Depending on the sample type (sample without gradient, sample with two zones and four zones), an appropriate set of models was prepared, consisting of hollow cylinders constituting individual zones and a cylinder which was the core of the sample. Each of the elements was exported to the stl format. In the Cura program, the elements were assembled (Figure 2) and their fillings were assigned in accordance with Table 1. The visualization of the models is presented in Figure 3 and Figure 4.

### 2.3. Preparation of PLA Samples

**Figure 5:**
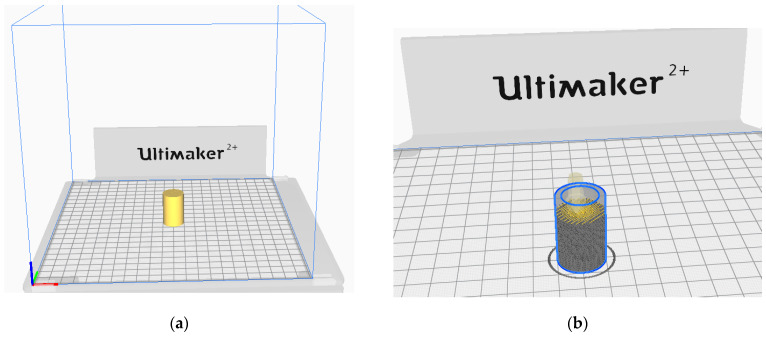
Arranging the sample on the platform in Cura program with the view of dividing into layers: (**a**) Positioning the sample on the worktable; (**b**) Layer split view.

The prepared models of the structures were produced based on the FDM technology on the Ultimaker 2+ printer. The printing parameters are summarized in Table 2, and an example of the model location on the working platform is shown in Figure 5.

**Figure 6:**
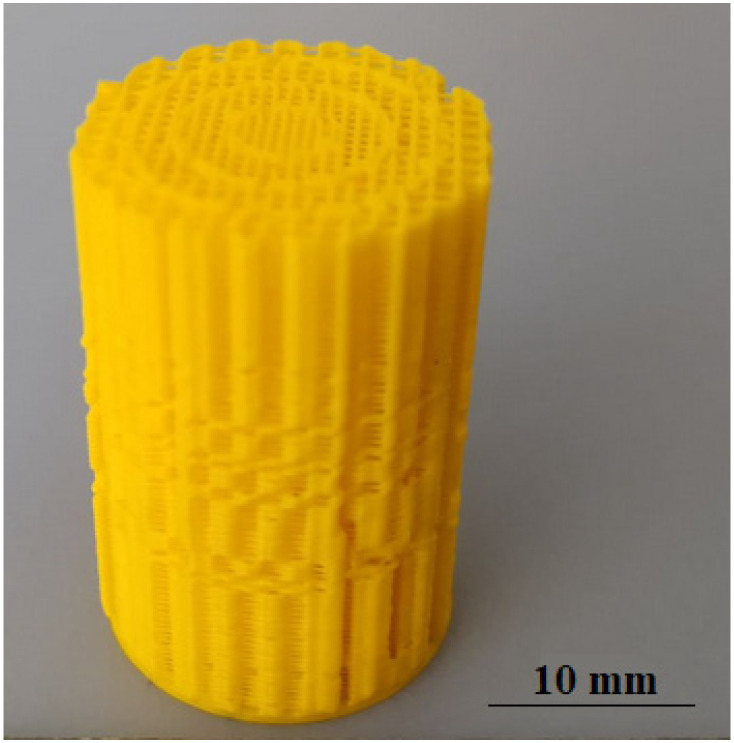
Sample printed from PLA.

Before the printing process, the glass platform was covered with a thin layer of glue to ensure a better adhesion of the sample to the glass printer platform. The platform was heated to a temperature of 60 °C. The printed samples were gently pulled off the platform after the platform had cooled down. Figure 6 shows an exemplary printed sample.

### 2.4. Computed Tomography Test

Computed tomography studies were performed on a Phoenix v|tome|x high-resolution X-ray scanner. It was possible to test samples with dimensions not exceeding 260 mm × 400 mm and weighing up to 10 kg. Two lamps were installed in the tomograph chamber, namely, a microfocus lamp (240 kV/320 W) and a nanofocus lamp (180 kV/15 W), which helped to obtain a resolution from 4 μm. However, the final resolution depended on the X-ray beam used, size, chemical composition and structure [30].

### 2.5. Static Compression Test

A compression test was carried out on the Instron 5982 universal testing machine with a maximum load of 100 kN. At the beginning of the study, the test conditions were defined in the Bluehill intron program. Parameters such as sample shape, sample size and the traverse feed speed were introduced. The speed of traverse during test was 2.5 mm min^−1^.

## 3. Results and Discussion

### 3.1. Assessment of the Spatial Architecture of PLA Samples by Computed Tomography

**Figure 7:**
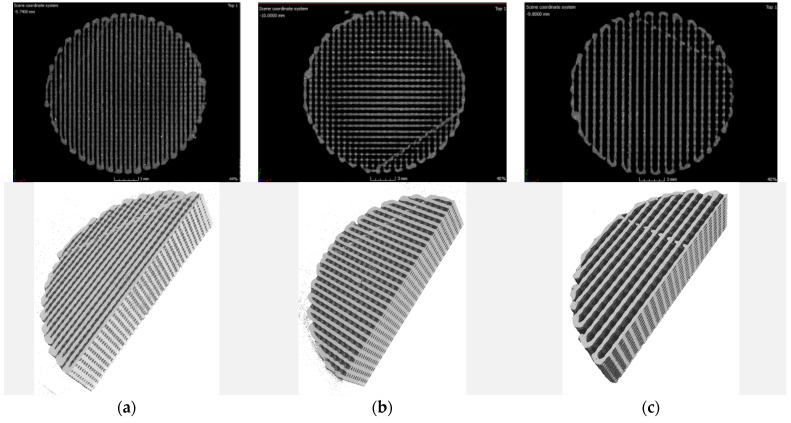
Exemplary images for single-zone (1S) samples with filling: (**a**) 60%; (**b**) 50%; (**c**) 40%.

**Figure 8:**
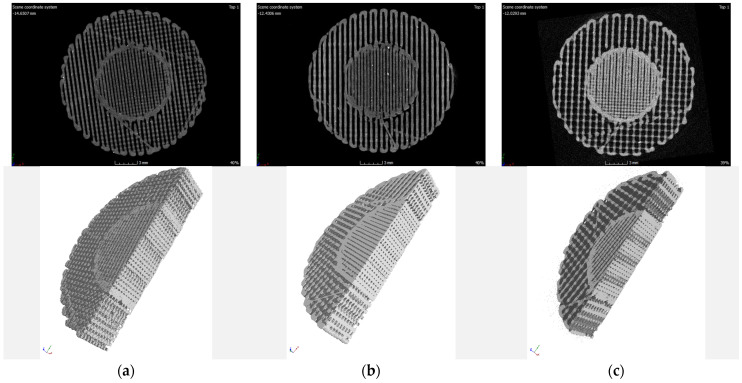
Exemplary images from the tomograph for two-zone (2S) samples: (**a**) 80–60%; (**b**) 80–50%; (**c**) 80–40%.

**Figure 9:**
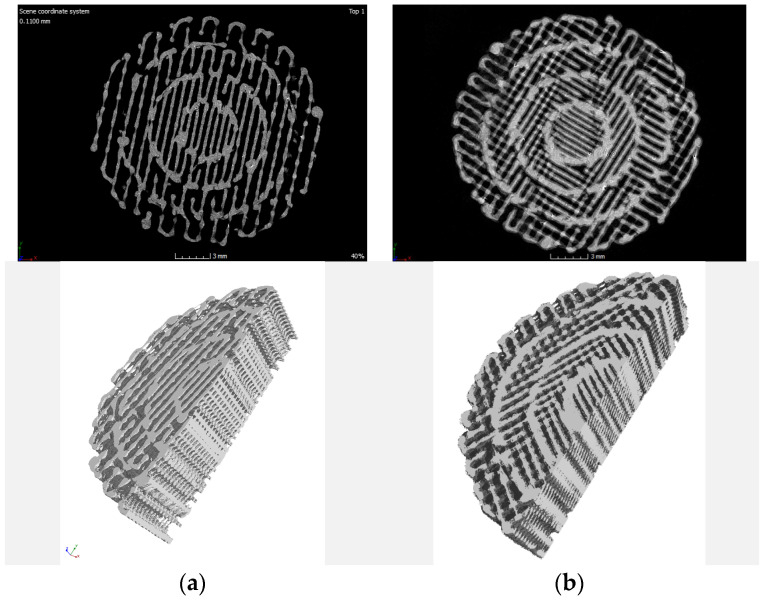
Exemplary images from the tomograph for four-zone (4S) samples: (**a**) 60–50–40–30%; (**b**) 70–60–50–40%.

The 3D images obtained from the tomograph allowed for a precise, spatial analysis of the examined structures in every direction and plane. Exemplary images recorded for the tested samples are shown in Figure 7, Figure 8 and Figure 9.

Based on the qualitative assessment of the obtained images, it can be concluded that the modelled spatial architecture of individual samples was correctly reproduced in the entire sample volume. Some samples show “threadlike” noise. It can be assumed that during the idle movement of the extruder, i.e., moving to the next point without printing, the filament escaped from the nozzle. To eliminate this effect, the printing temperature and/or retraction setting would have to be optimized in the future.

**Figure 10:**
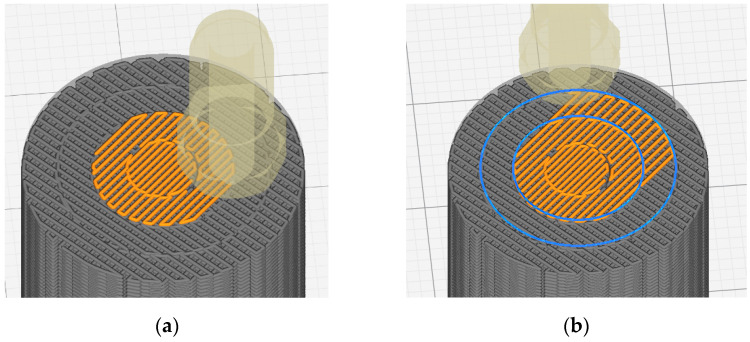
Build zones in gradient samples: (**a**) Two zones of one layer finished; (**b**) Start of printing the third zone.

In the case of PLA samples with a gradient structure, the accumulation of material at the boundary of individual zones is clearly visible. This is the result of the algorithm generated by the Cura program that controls the movement of the head. The zones were built one after the other, which causes the head to turn around the zone boundary twice, as schematically shown in Figure 10.

### 3.2. Assessment of Mechanical Properties in the Uniaxial Compression Test of PLA and PLA-T Samples Produced with the FDM Technique Depending on the Degree of Filling

**Figure 11:**
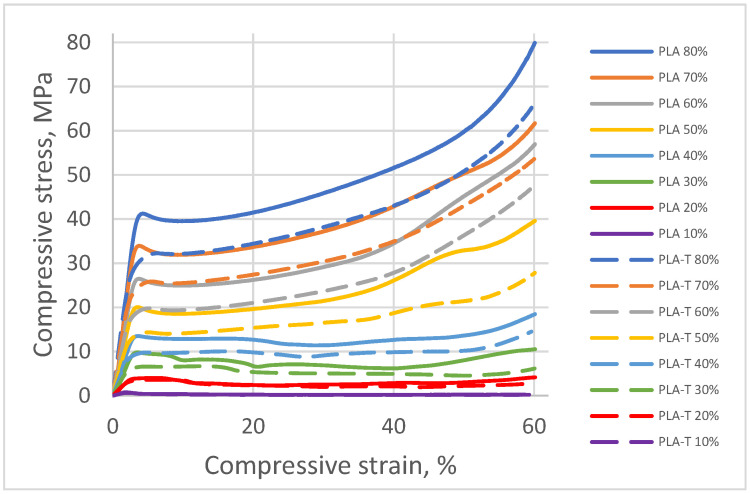
Compression curves for PLA and PLA-T samples without a fill gradient for 10, 20, 30, 40, 50, 60, 70, and 80% fillings.

The curves of compressive stress (σ) as a function of relative strain (ε) recorded for individual PLA, and PLA-T samples without a fill gradient for 10, 20, 30, 40, 50, 60, 70, and 80% fillings are presented in Figure 11.

**Figure 12:**
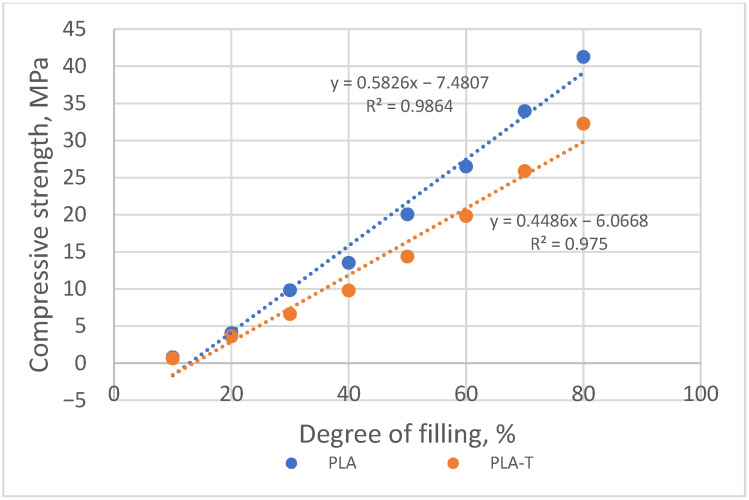
Relationship between the compressive strength and the filling degree of PLA and PLA-T samples.

According to the ASTM D1621 standard, the compressive strength is assumed to be equal to the pronounced yield point (if the yield point is 10% before deformation). In the absence of a clear yield point, the compressive strength was taken as the stress value at 10% deformation of the sample [31]. Based on the performed measurements, it was found that the compressive strength increased with an increase in the degree of filling of the samples for both PLA and PLA-T series (Figure 11). The samples with a filling degree of 80% showed the highest strength of 41.2 MPa for PLA and 32.3 MPa for PLA-T. The strength of the samples with the smallest filling degree was only 0.8 MPa for PLA and 0.6 MPa for PLA-T. The PLA-T samples showed lower compressive strength for each degree of filling than the corresponding PLA samples. All samples in both series (PLA and PLA-T) have a clear yield point. The relationship between the compressive strength and the degree of filling for both series of samples is linear, as shown in Figure 12.

**Figure 13:**
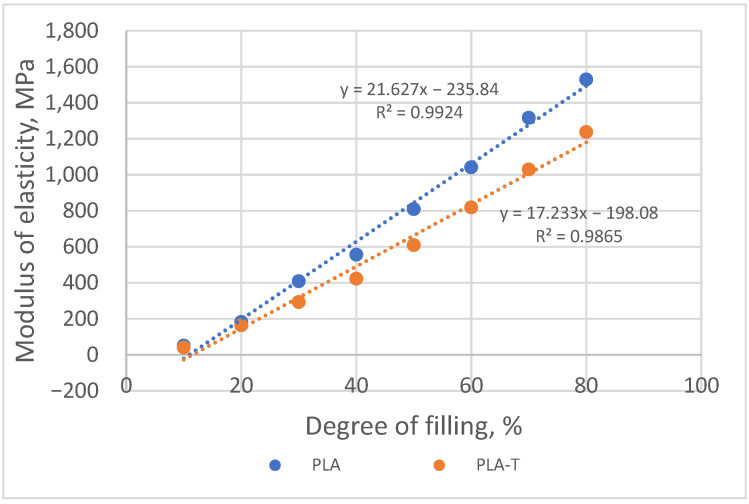
The relationship between modulus of elasticity in compression and the degree of filling of PLA and PLA-T samples.

Modulus of elasticity in compression for both series increased with increasing sample filling (Figure 13). The difference between the modulus values for both series of samples also increased from approx. 10 MPa for a 10% filling degree to approx. 290 MPa for a filling degree equal to 80%.

**Figure 14:**
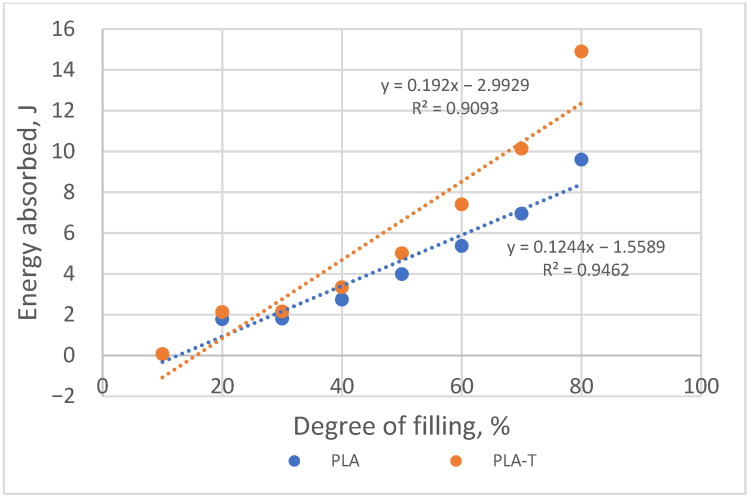
The relationship between the amount of energy absorbed by the sample during deformation and the degree of filling of PLA and PLA-T samples.

As the degree of filling increases, the amount of energy absorbed by the sample necessary for its deformation to a value corresponding to the compressive strength also increases (Figure 14). More energy was absorbed by the deformation of a sample made of PLA-T. At the same time, the difference between PLA and PLA-T decreased with a decrease in the degree of filling, and for samples with a filling below 30%, it was minimal.

### 3.3. Assessment of Mechanical Properties in the Uniaxial Compression Test of PLA and PLA-T Samples Produced with the FDM Technique with a Gradient Filling—Two Zones

**Figure 15:**
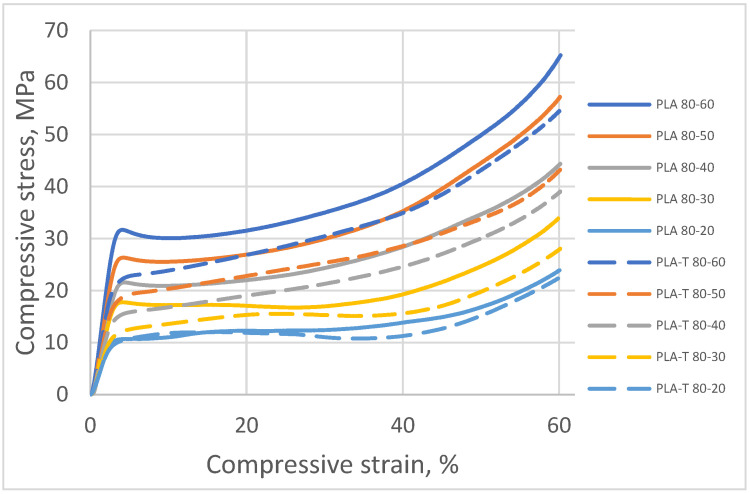
Compressive stress–strain diagram for PLA and PLA-T samples with a gradient filling (two zones).

The registered compressive stress–strain diagram for PLA and PLA-T samples with a gradient filling (two zones) are presented in Figure 15.

**Figure 16:**
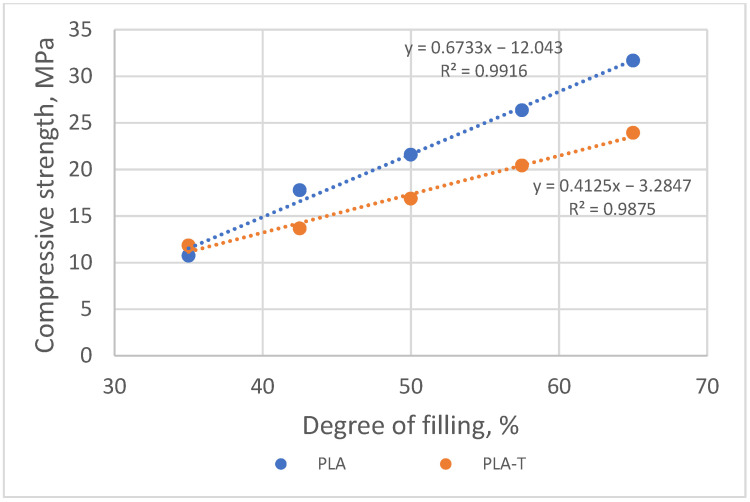
Relationship between compressive strength and sample filling degree of 2–zones PLA and PLA-T gradient samples.

Based on the measurements carried out for PLA and PLA-T samples, it was found that the compressive strength increased with an increase in the degree of filling in the second zone. The samples with the filling degree of 80–60% were characterized by the highest strength, amounting to 31.7 MPa for PLA and 23.9 MPa for PLA-T. The strength of the samples with the lowest filling degree of the second zone was 10.7 MPa for PLA and 11.8 MPa for PLA-T. For almost every degree of filling (without the lowest), the PLA-T samples had a lower compressive strength than the corresponding PLA samples. All of the samples in PLA series have a clear yield point, while the PLA-T samples demonstrated the absence of such a yield point. The relationship between the compressive strength and the degree of filling for both series of samples is linear, as shown in Figure 16.

**Figure 17:**
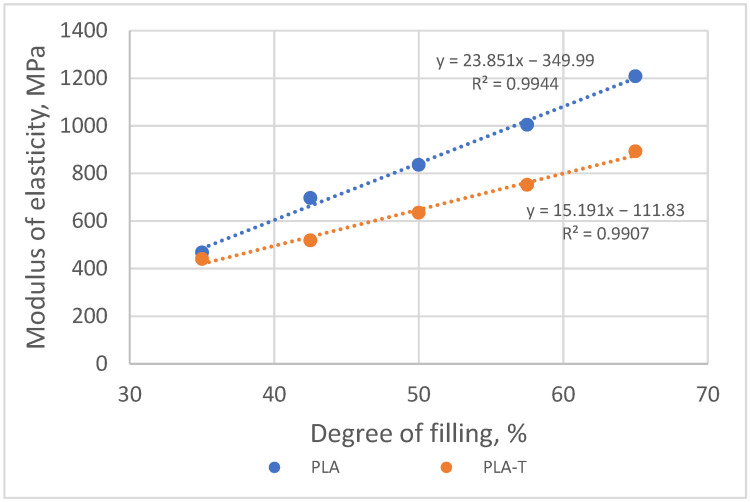
The relationship between modulus of elasticity in compression and the degree of filling of 2–zones PLA and PLA-T gradient samples.

Modulus of elasticity in compression for both series increased with an increasing sample degree of filling (Figure 17). The difference between the modulus values for both series of samples also increased from approx. 27 MPa for a 35% filling degree to approx. 315 MPa for a filling degree equal to 65%. Samples made of PLA-T showed a lower value of the modulus of elasticity than samples made of PLA.

**Figure 18:**
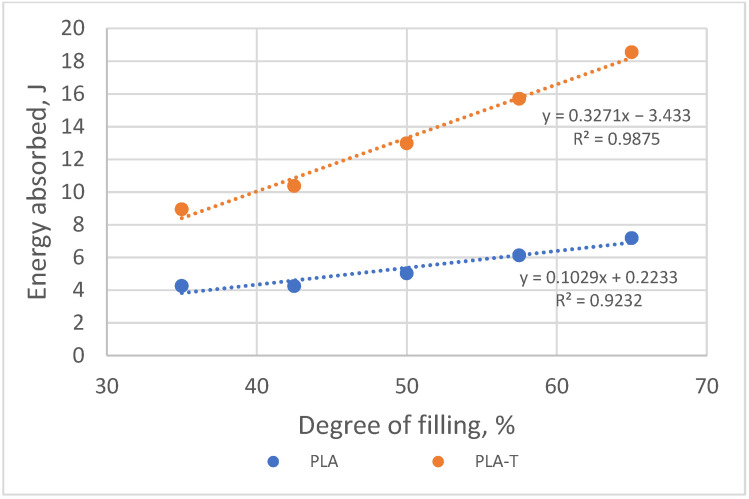
The relationship between the amount of energy absorbed by the sample during deformation and the degree of filling of 2–zones PLA and PLA-T gradient samples.

As the degree of filling increased, the amount of energy absorbed by the sample necessary for its deformation to a value corresponding to the compressive strength also increased (Figure 18). More energy was absorbed by the deformation of a sample made of PLA-T, while the difference between PLA and PLA-T decreased with a decrease in the degree of filling.

### 3.4. Assessment of Mechanical Properties in the Uniaxial Compression Test of PLA and PLA-T Samples Produced with the FDM Technique with Gradient Filling—Four Zones

**Figure 19:**
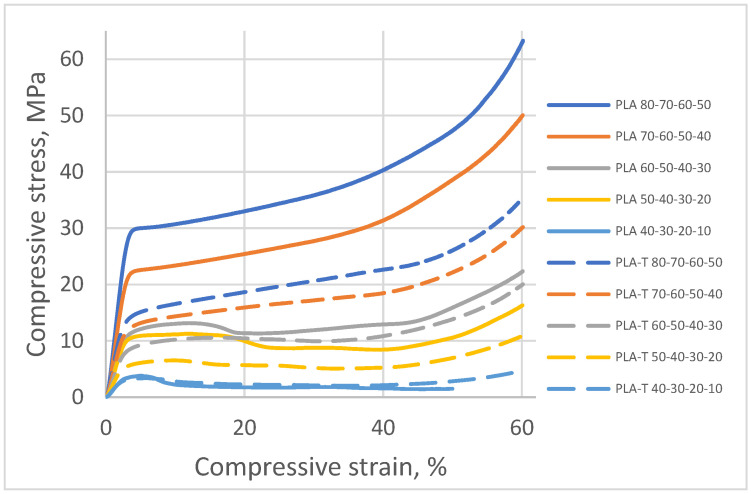
Compressive stress (σ) as a function of relative strain (ε) for PLA and PLA-T samples with a gradient filling (four zones).

The curves illustrating stress as a function of strain recorded for PLA and PLA-T samples with gradient filling (four zones) are presented in Figure 19.

**Figure 20:**
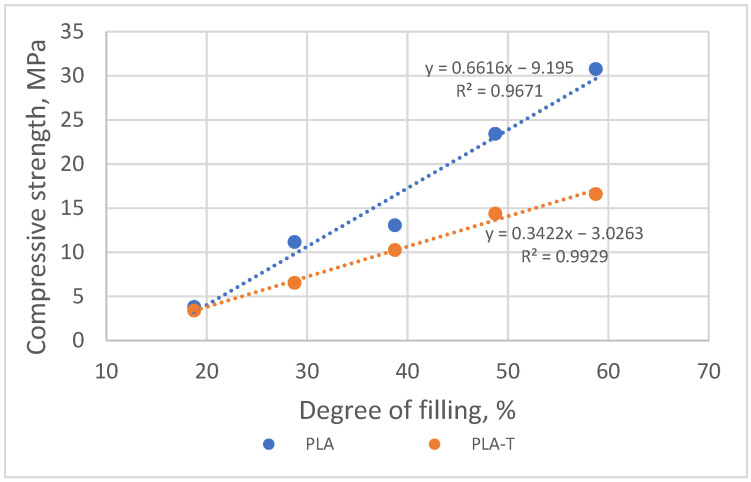
Relationship between compressive strength and sample filling degree of 4–zones PLA and PLA-T gradient samples.

When analyzing the obtained results, it can be noticed that similar to the previous series of samples (without and with 2-zones of porosity gradient), the compressive strength increased with the increase in the degree of filling in the fourth zone. The samples with a filling degree of 80–60–50–40% were characterized by the highest strength, amounting to 30.8 MPa for PLA and 16.6 MPa for PLA-T. The strength of the samples with the lowest filling degree of the fourth zone was 3.8 MPa and 3.4 MPa for PLA and PLA-T, respectively. The PLA-T samples presented lower compressive strength for each degree of filling than the corresponding PLA samples. A clear yield point was observed for the highest degrees of filling (the first four for PLA and the first three for PLA-T), while there is no clear yield point in other cases. As in the previously analyzed series, a linear relationship was found between the compressive strength and the porosity of the sample, which is shown in Figure 20.

**Figure 21:**
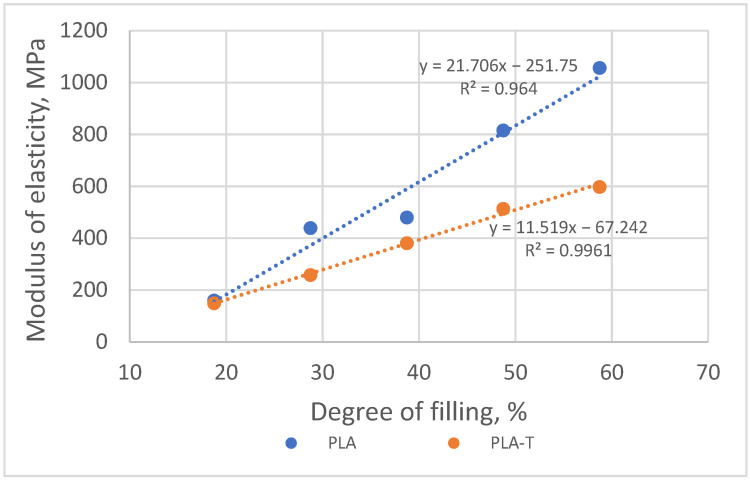
The relationship between modulus of elasticity in compression and the degree of filling of 4–zones PLA and PLA-T gradient samples.

Similar to the previous series of samples, the modulus of elasticity in compression for both series (PLA and PLA-T) increased with an increasing sample degree of filling (Figure 21). The difference between the modulus values for both series of samples also increased from approx. 10 MPa for a 18.75% filling degree to approx. 459 MPa for a filling degree equal to 58.75%. Samples made of PLA-T showed a lower value of the modulus of elasticity than samples made of PLA.

**Figure 22:**
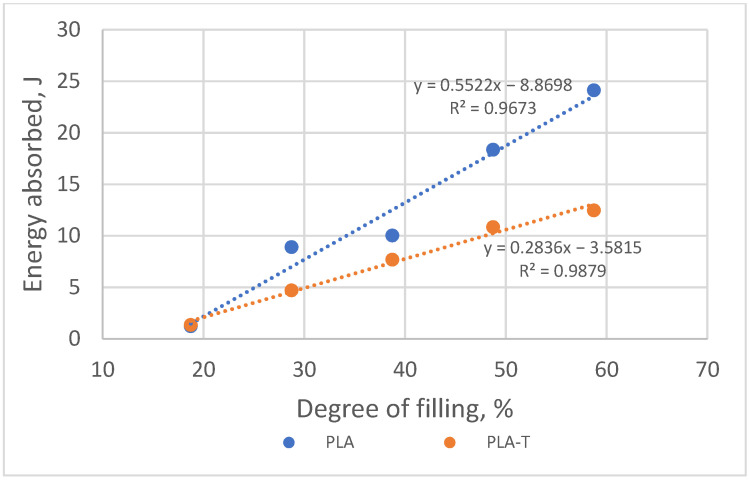
The relationship between the amount of energy absorbed by the sample during deformation and the degree of filling of 4–zones PLA and PLA-T gradient samples.

As the degree of filling increased, the amount of energy absorbed by the sample necessary for its deformation to a value corresponding to the compressive strength also increased (Figure 22). However, in contrast to the previous sample series, more energy was absorbed by the deformation of a sample made of PLA. The difference between PLA and PLA-T decreased with a decrease in the degree of filling, and for samples with a filling below 20% it is minimal.

**Figure 23:**
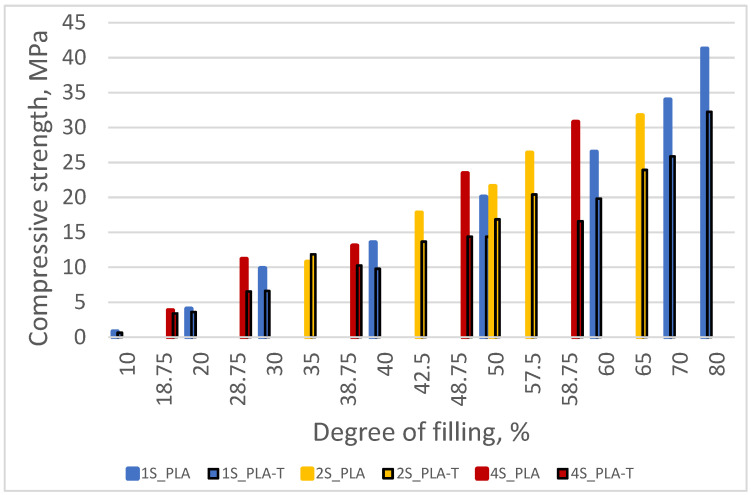
The relationship between compressive strength and the degree of filling for both materials (PLA and PLA-T) and all types (1S, 2S, 4S) of samples.

**Figure 24:**
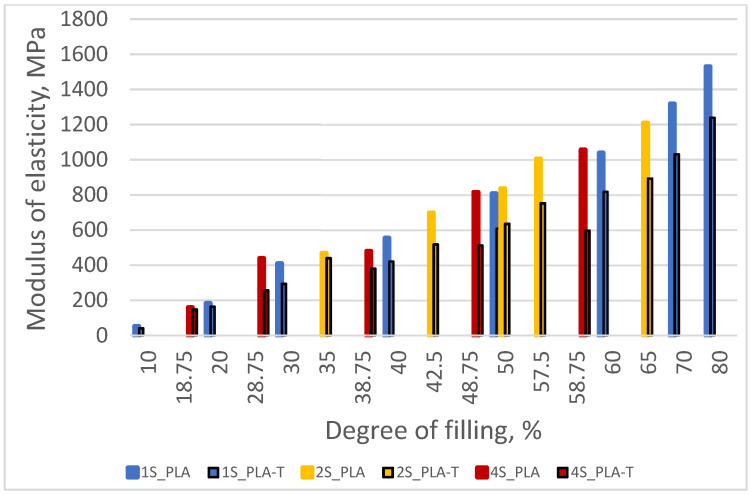
The relationship between modulus of elasticity in compression and the degree of filling for both materials (PLA and PLA-T) and all types (1S, 2S, 4S) of samples.

By analyzing the obtained results of compressive strength (Figure 23) and modulus of elasticity in compression (Figure 24) for all tested materials and types of samples, it was observed that the values of both parameters increased with an increasing degree of filling, while for a given degree of filling the samples made of pure PLA showed higher values.

**Figure 25:**
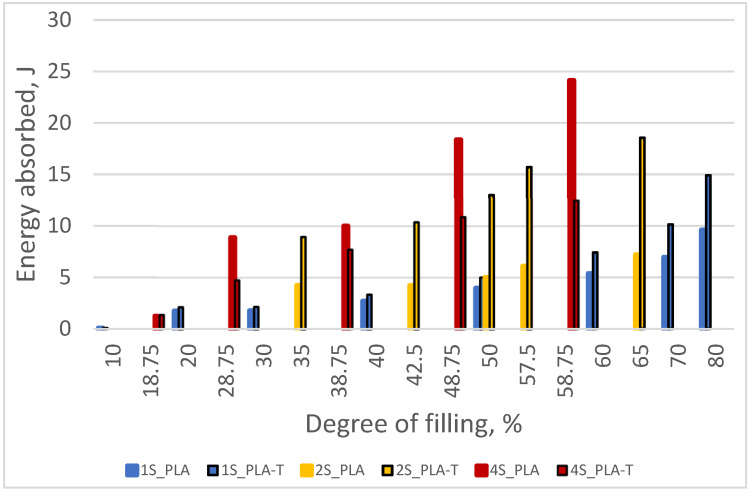
The relationship between the amount of energy absorbed by the sample during deformation and the degree of filling for both materials (PLA and PLA-T) and all types (1S, 2S, 4S) of samples.

A similar tendency can be observed in the case of energy absorbed by the sample during deformation (Figure 25), but in this case, for samples of type 1S and 2S, higher values were observed for samples made of PLA-T.

## 4. Conclusions

Based on the conducted research and an analysis of the obtained results, the following conclusions were formulated:

- The compressive strength increased with an increase in the degree of filling for all tested samples, while the samples made of pure PLA in each case showed higher values compared to the analogous samples made of PLA-T;
- The modulus of elasticity under compression increased with an increase in the degree of filling for all tested samples, while the differences in its values between the samples made of PLA and the corresponding samples of PLA-T were the greatest for the highest degree of filling and approached zero for the samples with the lowest density;
- The highest value of the modulus of elasticity in compression was obtained for the samples without the filling gradient of the highest density, while for the samples with the smallest filling degree, the highest value of the modulus was observed for the samples with a two-zone filling gradient;
- As the degree of filling increased, the value of the energy absorbed by all tested samples increased, while for samples without a porosity gradient and with a two-zone porosity gradient, higher values of energy absorbed by the sample were observed for those samples made of PLA-T compared to the corresponding samples made with PLA. For samples with a four-zone gradient, the PLA samples showed higher energy values. Higher values of energy absorbed by the sample may indicate greater resistance to dynamic loads, but this requires further research;
- The highest value of energy absorbed by the sample for the samples with the highest density was obtained for the architecture with a four-zone porosity gradient, while for the samples with the lowest degree of filling, the highest energy values were recorded for the two-zone porosity gradient.
- This study demonstrated that when using a simple method of dividing the geometry of a sample in a CAD program, it is possible to obtain a variable filling of the model at the stage of preparing the detail for printing in a free program (e.g., Cura). This makes it possible to control the mechanical properties of the samples across a wide range, while maintaining the lightness of the structure. In this work, however, only one type of infill pattern was used, i.e., the so-called line fill. Therefore, it seems advisable to extend the research in the future to multi-infill patterns and to verify the mechanical properties of the obtained samples, both in the tensile, compression and bending test. This would certainly contribute to a better understanding of the infill strategy on their mechanical properties. The authors are aware that the proposed methodology for modeling samples with a fill gradient is limited and its applicability in the case of complex geometries may be difficult and time-consuming. The best solution would be to develop our own gcode modification program, which, for example, based on the FEA analysis, adjusts the multi-infill, gradient pattern inside specimens depending on the stress distribution in the detail. The achievement topic fits within the most recent trend of 3D printing technology, which is developing rapidly, both on the national market and globally. Three-dimensional printing is part of the fourth industrial revolution, also comprising artificial intelligence, genetics, robotisation, medicine, and other areas, and is aimed at generating much smaller amounts of waste as compared with traditional production methods.

## Figures and Tables

**Table 1:** Types of generated 3D models.

1S Series—One zone	The Degree of Filling the Zones [%]		2S Series—One Zone	The Degree of Filling the Zones [%]	
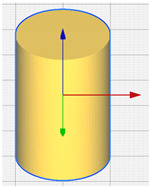	1	80%	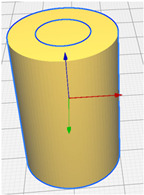	1	80–60%
	2	70%		2	80–50%
	3	60%		3	80–40%
	4	50%		4	80–30%
	5	40%		5	80–20%
	6	30%			
	7	20%			
	8	10%			
4S series—four zones	The degree of filling the zones [%]				
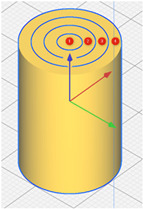	1	80–70–60–50%			
	2	70–60–50–40%			
	3	60–50–40–30%			
	4	50–40–30–20%			
	5	40–30–20–10%			

**Table 2:** 3D printing parameters.

Parameter	Value
Layer height	0.2 mm
Print speed	40 mm/s
Temperature	210 °C
Filling scheme	linear

## Data Availability

All data contained within the article.
